# A Comprehensive Analysis of Hungarian MODY Patients—Part II: Glucokinase MODY Is the Most Prevalent Subtype Responsible for about 70% of Confirmed Cases

**DOI:** 10.3390/life11080771

**Published:** 2021-07-30

**Authors:** Zsolt Gaál, Zsuzsanna Szűcs, Irén Kántor, Andrea Luczay, Péter Tóth-Heyn, Orsolya Benn, Enikő Felszeghy, Zsuzsanna Karádi, László Madar, István Balogh

**Affiliations:** 1 4th Department of Medicine, Jósa András Teaching Hospital, 4400 Nyíregyháza, Hungary; dr.gaal.zsolt@szszbmk.hu; 2 Division of Clinical Genetics, Department of Laboratory Medicine, Faculty of Medicine, University of Debrecen, 4032 Debrecen, Hungary; szucs.zsuzsanna@med.unideb.hu (Z.S.); madar.laszlo@med.unideb.hu (L.M.); 3 Department of Pediatrics, Jósa András Teaching Hospital, 4400 Nyíregyháza, Hungary; kantoriren@index.hu; 4 1st Department of Pediatrics, Semmelweis University, 1085 Budapest, Hungary; luczay.andrea@med.semmelweis-univ.hu (A.L.); toth-heyn.peter@med.semmelweis-univ.hu (P.T.-H.); 5 Department of Pediatrics, Szent György Hospital of Fejér County, 8000 Székesfehérvár, Hungary; bennorsolya@gmail.com (O.B.); zskaradi@mail.fmkorhaz.hu (Z.K.); 6 Department of Pediatrics, Faculty of Medicine, University of Debrecen, 4032 Debrecen, Hungary; felszeghy.eniko@med.unideb.hu

**Keywords:** MODY2, *GCK*-MODY, *GCK* mutations, Hungary

## Abstract

MODY2 is caused by heterozygous inactivating mutations in the glucokinase (*GCK*) gene that result in persistent, stable and mild fasting hyperglycaemia (5.6–8.0 mmol/L, glycosylated haemoglobin range of 5.6–7.3%). Patients with *GCK* mutations usually do not require any drug treatment, except during pregnancy. The *GCK* gene is considered to be responsible for about 20% of all MODY cases, transcription factors for 67% and other genes for 13% of the cases. Based on our findings, *GCK* and *HNF1A* mutations together are responsible for about 90% of the cases in Hungary, this ratio being higher than the 70% reported in the literature. More than 70% of these patients have a mutation in the *GCK* gene, this means that *GCK*-MODY is the most prevalent form of MODY in Hungary. In the 91 index patients and their 72 family members examined, we have identified a total of 65 different pathogenic (18) and likely pathogenic (47) *GCK* mutations of which 28 were novel. In two families, de novo *GCK* mutations were detected. About 30% of the *GCK*-MODY patients examined were receiving unnecessary OAD or insulin therapy at the time of requesting their genetic testing, therefore the importance of having a molecular genetic diagnosis can lead to a major improvement in their quality of life.

## 1. Introduction

### 1.1. GCK-MODY (MODY2)

MODY2 is caused by heterozygous inactivating mutations in the glucokinase (*GCK*) gene encoding a key regulator glycolytic enzyme of the hexokinase family [1]. It has two tissue-specific promoters and a different exon 1, the upstream promoter being functional in the pancreas (exon 1a) and brain, while the downstream one only in the liver (exons 1b and 1c), resulting in different isoforms of the *GCK* gene [2,3].

*GCK* has an important role in carbohydrate metabolism. It is responsible for the catalysis of the first reaction of the glycolytic pathway, the glucose phosphorylation [1]. *GCK* acts as a glucose sensor of the pancreatic beta-cells [1], therefore it is critical in the process of the regulation of insulin secretion and release.

In the case of *GCK*-MODY, a mildly elevated glucose level is caused by heterozygous loss-of-function mutations in the *GCK* gene. Any of the 10 exons and promoter of the pancreatic isoform of the *GCK* gene might be affected as no mutational hotspots have been identified. The mutations might affect enzyme kinetics or protein folding [4,5]. To date, almost 800 disease-causing small scale *GCK* mutations have been reported in the professional version of the HGMD (Human Gene Mutation Database, version 2021.1) associated with the MODY phenotype, the majority of them being missense alterations resulting in abnormal structure and/or function of the mutant protein, often affecting its kinetic parameters.

*GCK* gene mutations result in abnormal glucose sensing, raising the threshold of glucose-mediated insulin secretion. As a consequence, stable and mild fasting hyperglycaemia (5.6–8.0 mmol/L, glycosylated haemoglobin range of 5.6–7.3%) persists that does not deteriorate with age and is not associated with an increased risk of complications [6,7]. The clinical manifestation of *GCK*-MODY is generally nonprogressive, usually asymptomatic in childhood. The elevated glucose level is present from birth, therefore it is mostly detected incidentally [8,9]. Performing an oral glucose tolerance test (OGTT) can help to distinguish *GCK*-MODY patients from other types of MODY as in the case of *GCK*-MODY, patients generally have a small (<3.5 mmol/L) 2 h glucose increment [10].

Patients with *GCK* mutations usually do not require any drug treatment (except during pregnancy or in critical clinical situations), however, they often receive unnecessary insulin therapy or oral antidiabetic drug treatment [9]. Good glycaemic control can usually be achieved with only diet and exercise [11].

### 1.2. MODY Prevalence

The estimated MODY prevalence is around 1–5% of all diabetes mellitus cases, but it varies depending on the population studied [12,13]. The *GCK* gene is considered to be responsible for about 20% of all MODY cases, transcription factors for 67% and other genes for 13% of the cases [14]. *GCK* and *HNF1A* genes together are responsible for about 70% of all known MODY cases, the ratio of the two genes widely varying between countries [15]. For example in the United Kingdom, the prevalence of *GCK*-MODY is reported to be 32% [6,16], and 63% in the case of *HNF1A*-MODY [17]. The Norwegian MODY Registry reports a distribution of 53% *HNF1A*-MODY, 30% *GCK*-MODY, 7.5% *HNF4A*-MODY and 5.6% *HNF1B*-MODY [18]. A Polish study reports *GCK*-MODY to be the most prevalent with 83% [19] while the American SEARCH study reports *HNF1A*-MODY as the most prevalent form with roughly 60%, *GCK*-MODY being in the second position with 30% [20].

## 2. Materials and Methods

As this paper is Part II of two accompanying publications in the Journal, the patients and methods presented in this section are the same as the ones described in Part I of this article. The genes tested and genetic methods used during the study are presented in the Supplementary file (Part I of these articles).

### 2.1. Patients

A total of 450 unrelated index patients with suspected MODY diagnosis and their 202 family members have been referred to our laboratory for genetic testing from all around Hungary. All participants or their guardians have given informed consent to genetic testing according to national regulations.

### 2.2. Methods

Genomic DNA was isolated from peripheral blood leukocytes using the QIAamp Blood Mini kit (Qiagen GmbH, Hilden, Germany).

In the case of 102 index patients, Sanger sequencing of the *GCK*, *HNF1A* or *HNF4A* genes was performed using the BigDye Terminator v3.1 Cycle Sequencing kit (Applied Biosystems, Foster City, CA, USA) according to the manufacturer’s protocol.

Bidirectional pyrosequencing with a minimum coverage of 40× was performed on Roche GS Junior 454 pyrosequencing system (Roche 454 Life Sciences, Branford, CT, USA) in the case of 33 index patients.

The 311 index patient samples were sequenced on Illumina Miseq or NextSeq 550 (Illumina, San Diego, CA, USA) sequencer systems in 2 × 150 cycle (or 2 × 250 cycle in the case of the MODY MASTR kit) paired-end mode. Three different library preparation methods were used before sequencing. The MODY MASTR kit (Multiplicom, Niel, Belgium) was used to examine 7 genes in the case of 76 index patients. A custom-made and enrichment-based DNA library preparation kit (Qiagen, GmbH, Hilden, Germany) containing 17 genes was used in the case of 164 index patients, and another custom-designed gene panel (Twist Bioscience, South San Francisco, CA, USA) was used, examining 18 genes in the case of 69, and 20 genes in the case of 6 index patients. (Supplementary Table S1, see Part I) In the case of Illumina sequenced data, data analysis was performed using the NextGene software (SoftGenetics, State College, PA, USA).

MLPA (multiplex ligation-dependent probe amplification) was performed in the case of 32 index patients (as a single test in the case of 4 index patients and in addition to one of the above-mentioned methods in the case of 28 index patients) using SALSA MLPA Probemix P241 MODY Mix 1 and/or SALSA MLPA Probemix P357 MODY Mix 2 (MRC Holland, Amsterdam, Netherlands) according to the manufacturer’s protocol.

The testing method(s) used in the case of every index patient is described in the Supplementary Table S2 (Part I of these articles).

Cascade testing was performed in 202 family members usually by targeted Sanger sequencing of the respective exon of the MODY-causing gene in which their relative had a possibly pathogenic mutation.

### 2.3. Variant Confirmation

All variants obtained with next-generation sequencing that were suspected to be disease-causing were validated by Sanger sequencing. Furthermore, when the amplicon’s minimum coverage was <40× in the NGS data, the respective exons were also sequenced using the Sanger method.

### 2.4. Variant Filtering and Interpretation

All detected variants having a MAF > 0.01 (minor allele frequency) in the gnomAD population database were filtered. The remaining variants were classified according to the ACMG standards and guidelines [21,22]. A web-based interpretation tool, Franklin (Genoox) [23] was used to assist the classification. HGMD Professional and ClinVar databases were also used in variant interpretation.

### 2.5. Clinical Data Collection

Clinical data of patients and family members having a ‘pathogenic’ (‘P’) or ‘likely pathogenic’ (‘LP’) mutation in one of the MODY-causing genes was collected from their application form sent and filled out by their clinician at the time of requesting the genetic testing. The MODY probability calculator (https://www.diabetesgenes.org/, accessed on 20 March 2021) was used to calculate the probability of the patient having MODY when all the information required was available and the patient was under the age of 35, as the calculator cannot be used in case of patients older than that.

## 3. Results

GCK Mutations

From the 450 index patients examined, 132 tested positive for a pathogenic or likely pathogenic classified variant in one of the MODY-causing genes with a total of 89 mutations. *GCK* and *HNF1A* mutations together were responsible for about 90% of the cases, this ratio being higher in Hungary than the 70% reported in the literature [15]. More than 70% (65/89) of the mutations among the index patients were found in the *GCK* gene (Table 1). With targeted cascade testing of family members, we identified an additional 95 positive cases, resulting in a total of 227 patients with a molecular genetic diagnosis of MODY. More than 70% of these patients have a mutation in the *GCK* gene, which means that *GCK*-MODY is the most prevalent form of MODY in Hungary.

**Figure 1:**
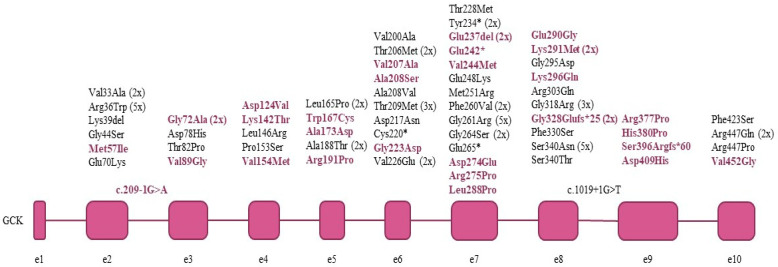
*GCK* mutations detected in the index patients. Novel mutations are shown in colour.

In the 91 index patients and their 72 family members, we have identified a total of 65 different pathogenic (18) and likely pathogenic (47) *GCK* mutations, summarized in Table 2 and Figure 1. Every mutation detected was in heterozygous form. Eighteen mutations were found in more than one apparently unrelated families, the most frequent ones being p.Arg36Trp (5 families), p.Gly261Arg (G > A 5 families and G > C 1 family) and p.Ser340Asn (5 families). Of the detected mutations, 40% (28/65) are novel, while 60% (37/65) have been previously described in the literature. Almost 85% (55/65) of the detected *GCK* mutations were missense mutations resulting in an amino acid change. In addition, four such mutations were found at exon/intron boundaries of the coding sequence, possibly disrupting exon splicing as well (Table 3).

In the case of two families (F101, F173), the p.Ala188Thr and p.Val226Glu mutations were both detected and co-segregated in the proband and her parent, suggesting a cis position.

Table 4 presents the clinical data of the index patients and their family members. Obesity is not characteristic of these patients, only about 10% of them have their BMI out of the range considered healthy. The age of diagnosis differs widely among the patients, and they have generally received their molecular genetic diagnosis of MODY several years after their diagnosis of diabetes. We had information regarding their treatment in 125 cases. Almost half of the patients examined do not receive any treatment or control their blood sugar levels only by maintaining a healthy diet, which is in accordance with the literature. However, around 10% of these patients receive unnecessary insulin treatment and another 16% are on some oral antidiabetic drug, also unnecessary (Table 5). Their HbA1c levels are generally around 7.0% or lower.

The detected *GCK* mutation was shown to be de novo in two cases (Table 4, F041 and F375).

We had enough information to use the MODY calculator in about half of the patients. In the case of about three-quarters of these patients (62/81), the calculator showed a 75.5% probability of the patient having MODY, this was the highest probability we could get using the calculator.

## 4. Discussion

Two hundred and twenty-seven patients were diagnosed with MODY in our examined cohort from all over Hungary in about 10 years with a 70% mutation rate in the *GCK* gene, meaning that the most prevalent form of monogenic diabetes in Hungary is the *GCK*-MODY.

Although *GCK*-MODY patients generally do not need any treatment, around 30% of the patients examined were receiving an unnecessary OAD or insulin therapy. We would like to emphasize once again the importance of having a proper molecular genetic diagnosis, as this can lead to a major improvement in the patients’ quality of life by stopping their drug treatment. 

The majority of the examined patients had an HbA1c level of 7.0% or lower, this being in accordance with the mildly elevated level reported in the literature and in contrast with the *HNF1A* patients we examined, where about 60% of the patients had a value of 7.0% or above.

As two families had de novo *GCK* mutations, one criterion of MODY about the transgenerational occurrence of the disease should be treated with caution—the lack of apparent inheritance pattern does not exclude the possibility of having a MODY.

The effect of the pathogenic and likely pathogenic mutations on the kinetics of the glucokinase enzyme is still not precisely known in many cases, therefore we plan to further investigate this question in the future.

## Figures and Tables

**Table 1:** Number of patients harbouring a pathogenic/likely pathogenic mutation in one of the MODY-causing genes.

Gene	No. of Index Patients with ‘P’/’LP’ Mutations	No. of Index Patients and Their Family Members with ‘P’/’LP’ Mutations
*GCK*	91 (68.9%)	163 (71.8%)
*HNF1A*	30 (22.7%)	48 (21.1%)
other MODY-causing gene ^1^	11 (8.3%)	16 (7.0%)
Total	132 (100.0%)	227 (100.0%)

^1^ *ABCC8*, *HNF1B*, *HNF4A*, *INS*, *KCNJ11*. P: pathogenic; LP: likely pathogenic.

**Table 2:** Pathogenic (18) and likely pathogenic (47) mutations in the *GCK* gene.

Nucleotide Change	Protein Change	Exon/Intron	Function	ACMG	ACMG Evidence	ClinVar	gnomAD Alleles (MAF)	Pr/FM	Family ID	Novel/Known	Reference
c.98T > C	p.Val33Ala	exon 2	Missense	Pathogenic	PM1 (2); PM2 (2); PM5 (1); PP2 (1); PP3 (1); PP5 (3)	Pathogenic (1)	N/A	2/0	F306, F454	known	[24]
c.106C > T	p.Arg36Trp	exon 2	Missense	Pathogenic	PM1 (2); PM2 (2); PM5 (1); PP1 (2); PP2 (1); PP3 (1); PP5 (3)	Pathogenic/ Likely pathogenic (4)	4 (0.00001414)	5/4	F028, F105, F310, F433, F434	known	[25]
c.115_117delAAG	p.Lys39del	exon 2	In-Frame	Pathogenic	PM1 (2); PM2 (2); PM4 (2); PP1 (3)	N/A	N/A	1/2	F044	known	[26]
c.130G > A	p.Gly44Ser	exon 2	Missense	Pathogenic	PM1 (2); PM2 (2); PM5 (1); PP1 (3); PP2 (1); PP3 (1); PP5 (3)	Pathogenic (1)	N/A	1/3	F133	known	[27]
**c.171G > T**	**p.Met57Ile**	**exon 2**	**Missense**	**Pathogenic**	**PM1 (2); PM2 (2); PP2 (1); PP3 (1); PP5 (3); PS1 (1)**	**Pathogenic (1)**	**N/A**	**1/0**	**F463**	**novel**	
c.208G > A	p.Glu70Lys	exon 2	Missense/Splicing	Likely pathogenic	PM1 (2); PM2 (2); PP2 (1); PP3 (1); PP5 (1)	N/A	N/A	1/0	F389	known	[28]
**c.209-1G > A**	**Splice**	**intron 2**	**Splicing**	**Likely pathogenic**	**PM2 (2); PVS1 (4)**	**N/A**	**1 (0.00003186)**	**1/0**	**F499**	**novel**	
**c.215G > C**	**p.Gly72Ala**	**exon 3**	**Missense**	**Likely pathogenic**	**PM1 (2); PM2 (2); PM5 (2); PP1 (2); PP2 (1); PP3 (1)**	**N/A**	**N/A**	**2/3**	**F069, F274**	**novel**	
c.232G > C	p.Asp78His	exon 3	Missense	Likely pathogenic	PM1 (2); PM2 (2); PP2 (1); PP3 (1)	N/A	N/A	1/3	F006	known	[29]
c.244A > C	p.Thr82Pro	exon 3	Missense	Likely pathogenic	PM1 (2); PM2 (2); PM5 (1); PP2 (1); PP3 (1)	N/A	N/A	1/1	F472	known	[30]
**c.266T > G**	**p.Val89Gly**	**exon 3**	**Missense**	**Likely pathogenic**	**PM1 (2); PM2 (2); PP2 (1); PP3 (1)**	**N/A**	**N/A**	**1/1**	**F213**	**novel**	
**c.371A > T**	**p.Asp124Val**	**exon 4**	**Missense**	**Likely pathogenic**	**PM1 (2); PM2 (2); PM5 (1); PP1 (1); PP2 (1); PP3 (1); PP5 (2)**	**Likely pathogenic (1)**	**N/A**	**1/1**	**F083**	**novel**	
**c.425A > C**	**p.Lys142Thr**	**exon 4**	**Missense**	**Likely pathogenic**	**PM1 (2); PM2 (2); PP2 (1); PP3 (1)**	**N/A**	**N/A**	**1/0**	**F250**	**novel**	
c.437T > G	p.Leu146Arg	exon 4	Missense	Likely pathogenic	PM1 (2); PM2 (2); PP2 (1); PP3 (1)	N/A	N/A	1/0	F018	known	[31]
c.457C > T	p.Pro153Ser	exon 4	Missense	Likely pathogenic	PM1 (2); PM2 (2); PP1 (1); PP2 (1); PP3 (1)	Uncertain significance (2)	N/A	1/1	F103	known	[32]
**c.460G > A**	**p.Val154Met**	**exon 4**	**Missense**	**Likely pathogenic**	**PM1 (2); PM2 (2); PP1 (2); PP2 (1); PP3 (1)**	**N/A**	**N/A**	**1/1**	**F381**	**novel**	
c.494T > C	p.Leu165Pro	exon 5	Missense	Likely pathogenic	PM1 (2); PM2 (2); PP2 (1); PP3 (1)	N/A	N/A	2/0	F092, F291	known	[33]
**c.501G > C**	**p.Trp167Cys**	**exon 5**	**Missense**	**Likely pathogenic**	**PM1 (2); PM2 (2); PP2 (1); PP3 (1)**	**N/A**	**N/A**	**1/0**	**F349**	**novel**	
**c.518C > A**	**p.Ala173Asp**	**exon 5**	**Missense**	**Likely pathogenic**	**PM1 (2); PM2 (2); PP2 (1); PP3 (1)**	**N/A**	**N/A**	**1/0**	**F002**	**novel**	
c.562G > A	p.Ala188Thr	exon 5	Missense	Pathogenic	PM1 (2); PM2 (2); PM5 (1); PP1 (2); PP2 (1); PP3 (1); PP5 (3)	Pathogenic (2)	1 (0.000003982)	2/2	F101, F173	known	[34]
**c.572G > C**	**p.Arg191Pro**	**exon 5**	**Missense**	**Likely pathogenic**	**PM1 (2); PM2 (2); PM5 (2); PP2 (1); PP3 (1)**	**N/A**	**N/A**	**1/0**	**F374**	**novel**	
c.599T > C	p.Val200Ala	exon 6	Missense	Likely pathogenic	PM1 (2); PM2 (2); PP2 (1); PP3 (1)	N/A	N/A	1/0	F094	known	[10]
c.617C > T	p.Thr206Met	exon 6	Missense	Likely pathogenic	PM1 (2); PM2 (2); PM5 (2); PP1 (2); PP2 (1); PP3 (1); PP5 (1)	N/A	1 (0.000003977)	2/2	F031, F116	known	[35]
**c.620T > C**	**p.Val207Ala**	**exon 6**	**Missense**	**Likely pathogenic**	**PM1 (2); PM2 (2); PP2 (1); PP3 (1)**	**N/A**	**N/A**	**1/0**	**F316**	**novel**	
**c.622G > T**	**p.Ala208Ser**	**exon 6**	**Missense**	**Likely pathogenic**	**PM1 (2); PM2 (2); PM5 (1); PP2 (1); PP3 (1)**	**N/A**	**N/A**	**1/0**	**F205**	**novel**	
c.623C > T	p.Ala208Val	exon 6	Missense	Pathogenic	PM1 (2); PM2 (2); PM5 (1); PP1 (3); PP2 (1); PP3 (1)	Uncertain significance (1)	1 (0.000003977)	1/2	F227	known	[36]
c.626C > T	p.Thr209Met	exon 6	Missense	Pathogenic	PM1 (2); PM2 (2); PP1 (2); PP2 (1); PP3 (1); PP5 (3)	Pathogenic (1)	N/A	3/1	F041, F042, F153	known	[25]
c.649G > A	p.Asp217Asn	exon 6	Missense	Likely pathogenic	BS4 (1); PM1 (2); PM2 (2); PP2 (1); PP3 (1)	Uncertain significance (4)	1	1/0	F150	known	[37]
c.660C > A	p.Cys220*	exon 6	Nonsense	Likely pathogenic	PM2 (2); PVS1 (4)	N/A	N/A	1/0	F165	known	[38]
**c.668G > A**	**p.Gly223Asp**	**exon 6**	**Missense**	**Likely pathogenic**	**PM1 (2); PM2 (2); PM5 (2); PP2 (1); PP3 (1)**	**N/A**	**N/A**	**1/2**	**F411**	**novel**	
c.677T > A	p.Val226Glu	exon 6	Missense	Likely pathogenic	PM1 (2); PM2 (2); PM5 (2); PP1 (2); PP2 (1); PP3 (1)	N/A	N/A	2/2	F101, F173	known	[39]
c.683C > T	p.Thr228Met	exon 7	Missense	Pathogenic	PM1 (2); PM2 (2); PM5 (1); PP2 (1); PP3 (1); PP5 (3)	Pathogenic (5)	1 (0.000003999)	1/0	F065	known	[40]
c.702C > A	p.Tyr234*	exon 7	Nonsense	Pathogenic	PM2 (2); PP1 (2); PVS1 (4)	N/A	N/A	2/2	F011, F244	known	[41]
**c.709_711delGAG**	**p.Glu237del**	**exon 7**	**In-frame**	**Likely pathogenic**	**PM1 (2); PM2 (2); PM4 (2); PP1 (2)**	**N/A**	**N/A**	**2/1**	**F070, F246**	**novel**	
**c.724G > T**	p.Glu242*	**exon 7**	**Nonsense**	**Pathogenic**	**PM2 (2); PP1 (3); PVS1 (4)**	**N/A**	**N/A**	**1/3**	**F382**	**novel**	
**c.730G > A**	**p.Val244Met**	**exon 7**	**Missense**	**Likely pathogenic**	**PM1 (2); PM2 (2); PP2 (1); PP3 (1); PP5 (1)**	**Conflicting** **(1 LP, 1 VUS)**	**N/A**	**1/0**	**F400**	**novel**	
c.742G > A	p.Glu248Lys	exon 7	Missense	Likely pathogenic	PM1 (2); PM2 (2); PP2 (1); PP3 (1); PP5 (1)	N/A	1 (0.000003982)	1/0	F219	known	[42]
c.752T > G	p.Met251Arg	exon 7	Missense	Pathogenic	PM1 (2); PM2 (2); PM5 (1); PP1 (3); PP2 (1); PP3 (1)	N/A	N/A	1/4	F145	known	[10]
c.778T > G	p.Phe260Val	exon 7	Missense	Likely pathogenic	PM1 (2); PM2 (2); PM5 (1); PP2 (1); PP3 (1)	N/A	N/A	2/2	F408, F455	known	[43]
c.781G > A	p.Gly261Arg	exon 7	Missense	Pathogenic	PM1 (2); PM2 (2); PP1 (1); PP2 (1); PP3 (1); PP5 (3); PS1 (3)	Pathogenic (4)	1 (0.000003983)	4/1	F126, F191, F202, F272	known	[40]
c.781G > C	p.Gly261Arg	exon 7	Missense	Pathogenic	PM1 (2); PM2 (2); PP2 (1); PP3 (1); PP5 (3); PS1 (3)	Pathogenic (1)	N/A	1/0	F108	known	[44]
c.790G > A	p.Gly264Ser	exon 7	Missense	Likely pathogenic	PM1 (2); PM2 (2); PP2 (1); PP3 (1); PP5 (1)	Pathogenic (1)	N/A	2/0	F080, F216	known	[35]
c.793G > T	p.Glu265*	exon 7	Nonsense	Pathogenic	PM2 (2); PP1 (1); PP5 (3); PVS1 (4)	Pathogenic (2)	N/A	1/1	F035	known	[45]
**c.822C > A**	**p.Asp274Glu**	**exon 7**	**Missense**	**Likely pathogenic**	**PM1 (2); PM2 (2); PP2 (1); PP3 (1)**	**N/A**	**1 (0.000003992)**	**1/0**	**F280**	**novel**	
**c.824G > C**	**p.Arg275Pro**	**exon 7**	**Missense**	**Likely pathogenic**	**PM1 (2); PM2 (2); PM5 (1); PP2 (1); PP3 (1)**	**N/A**	**N/A**	**1/0**	**F107**	**novel**	
**c.863T > C**	**p.Leu288Pro**	**exon 7**	**Missense/splicing**	**Likely pathogenic**	**PM1 (2); PM2 (2); PP2 (1); PP3 (1)**	**N/A**	**N/A**	**1/1**	**F403**	**novel**	
**c.869A > G**	**p.Glu290Gly**	**exon 8**	**Missense**	**Likely pathogenic**	**PM1 (2); PM2 (2); PP1 (2); PP2 (1); PP3 (1)**	**N/A**	**N/A**	**1/0**	**F375**	**novel**	
**c.872A > T**	**p.Lys291Met**	**exon 8**	**Missense**	**Pathogenic**	**PM1 (2); PM2 (2); PM5 (1); PP1 (3); PP2 (1); PP3 (1)**	**N/A**	**N/A**	**2/6**	**F043, F046**	**novel**	
c.884G > A	p.Gly295Asp	exon 8	Missense	Likely pathogenic	PM1 (2); PM2 (2); PP1 (1); PP2 (1); PP3 (1)	N/A	N/A	1/1	F038	known	[39]
**c.886A > C**	**p.Lys296Gln**	**exon 8**	**Missense**	**Likely pathogenic**	**PM1 (2); PM2 (2); PP2 (1); PP3 (1)**	**N/A**	**2 (0.000008008)**	**1/0**	**F296**	**novel**	
c.908G > A	p.Arg303Gln	exon 8	Missense	Likely pathogenic	PM1 (2); PM2 (2); PM5 (1); PP2 (1); PP3 (1); PP5 (2)	Likely pathogenic (1)	N/A	1/1	F167	known	[46]
c.952G > A	p.Gly318Arg	exon 8	Missense	Pathogenic	PM1 (2); PM2 (2); PM5 (1); PP1 (3); PP2 (1); PP3 (1); PP5 (3)	Pathogenic (1)	N/A	3/5	F016, F209, F353	known	[47]
**c.982delG**	p.Gly328Glufs*25	**exon 8**	**Frameshift**	**Pathogenic**	**PM2 (2); PP1 (3); PVS1 (4)**	**N/A**	**N/A**	**2/5**	**F085, F162**	**novel**	
c.989T > C	p.Phe330Ser	exon 8	Missense	Likely pathogenic	PM1 (2); PM2 (2); PP1 (1); PP2 (1); PP3 (1)	Uncertain significance (1)	N/A	1/1	F122	known	[48]
c.1019G > A	p.Ser340Asn	exon 8	Missense/ splicing	Likely pathogenic	PM1 (2); PM2 (2); PM5 (1); PP1 (2); PP2 (1); PP3 (1)	N/A	N/A	5/4	F027, F062, F163, F187, F197	known	[32]
c.1019G > C	p.Ser340Thr	exon 8	Missense/ splicing	Likely pathogenic	PM1 (2); PM2 (2); PM5 (1); PP1 (1); PP2 (1); PP3 (1)	N/A	N/A	1/1	F435	known	[49]
c.1019 + 1G > T	Splice	intron 8	Splicing	Likely pathogenic	PM2 (2); PVS1 (3)	N/A	N/A	1/0	F131	known	[50]
**c.1130G > C**	**p.Arg377Pro**	**exon 9**	**Missense**	**Likely pathogenic**	**PM1 (2); PM2 (2); PM5 (2); PP2 (1); PP3 (1)**	**N/A**	**N/A**	**1/0**	**F482**	**novel**	
**c.1139A > C**	**p.His380Pro**	**exon 9**	**Missense**	**Likely pathogenic**	**PM1 (2); PM2 (2); PP1 (1); PP2 (1); PP3 (1)**	**N/A**	**N/A**	**1/1**	**F200**	**novel**	
**c.1186_1193delAGCCGCAG**	p.Ser396Argfs*60	**exon 9**	**Frameshift**	**Likely pathogenic**	**PM2 (2); PVS1 (4)**	**N/A**	**N/A**	**1/0**	**F314**	**novel**	
**c.1225G > C**	**p.Asp409His**	**exon 9**	**Missense**	**Likely pathogenic**	**PM1 (2); PM2 (2); PP1 (1); PP2 (1); PP3 (1)**	**N/A**	**N/A**	**1/1**	**F113**	**novel**	
c.1268T > C	p.Phe423Ser	exon 10	Missense	Likely pathogenic	PM1 (2); PM2 (2); PM5 (1); PP2 (1); PP3 (1)	Uncertain significance (1)	N/A	1/0	F275	known	[51]
c.1340G > A	p.Arg447Gln	exon 10	Missense	Likely pathogenic	PM1 (2); PM2 (2); PM5 (1); PP1 (2); PP2 (1); PP3 (1); PP5 (2)	Likely pathogenic (1)	N/A	2/2	F201, F373	known	[29,42]
c.1340G > C	p.Arg447Pro	exon 10	Missense	Likely pathogenic	PM1 (2); PM2 (2); PM5 (1); PP2 (1); PP3 (1)	Uncertain significance (1)	N/A	1/0	F273	known	[52]
**c.1355T > G**	**p.Val452Gly**	**exon 10**	**Missense**	**Likely pathogenic**	**PM1 (2); PM2 (2); PP2 (1); PP3 (1)**	**N/A**	**N/A**	**1/0**	**F263**	**novel**	

*GCK* reference sequence: NM_000162.5, novel mutations are shown in bold. ACMG: shows the classification of the mutation based on the ACMG guidelines; ACMG evidence: the criteria and their strength used for the ACMG classification, as follows: (1)—supporting, (2)—moderate, (3)—strong, (4)—very strong, (5)—stand-alone; ClinVar: the classification of the mutation according to ClinVar, with the number of submissions in brackets; gnomAD MAF: minor allele frequency of the mutation in the gnomAD database; Pr/FM: number of probands/their family members the mutation was found in; family ID: identification of the families the mutation was found in.

**Table 3:** *GCK* mutations distributed by the amino acid consequence.

Consequence	No. of Mutations
Missense	51 (78.5%)
Missense and/or splicing	4 (6.2%)
Splicing	21 (3.1%)
Nonsense	4 (6.2%)
Frameshift	2 (3.1%)
In-frame	2 (3.1%)

**Table 4:** Clinical data of patients with *GCK* mutation.

Family ID	Sample ID	Age at Diagnosis of Diabetes	Age at Receiving Genetic Dg	BMI *	Obesity	Complications	Therapy BEFORE Genetic Diagnosis	FPG (0′) (mmol/L)	PPG (120′) (mmol/L)	HbA1c % (mmol/mol)	MODY Calculator (%)	Family Screening
**F002**	**P002**	**32**	**47**	**26.2**	**no**	**none**	**OAD—metformin**	**7.3**	**N/A**	**6.3 (45.4)**	**15.1**	**no family members tested**
**F006**	**P015**	**31**	**N/A**	**23**	**no**	**none**	**insulin**	**6.8**	**19.0**	**6.7 (49.7)**	**12.6**	**multiple generations affected**
F006	P016	46	N/A	33	yes	IHD, PAD	insulin	7.0	12.0	8.2 (66.1)	N/A	multiple generations affected
F006	P017	3	4	15.6	no	none	diet	6.0	9.0	N/A	N/A	multiple generations affected
F006	P018	no diabetes	1	N/A	N/A	N/A	N/A	N/A	N/A	N/A	N/A	multiple generations affected
**F011**	**P028**	**17**	**27**	**19.8**	**no**	**none**	**OAD—acarbose**	**7.0**	**6.0**	**6.2 (44.3)**	**75.5**	**multiple generations affected**
F011	P029	42	53	24.7	no	none	OAD— sulphonylurea	9.2	5.2	6.5 (47.5)	N/A	multiple generations affected
F011	P030	2	3	15.4	no	none	diet	6.7	N/A	N/A	N/A	multiple generations affected
**F016**	**P037**	**childhood**	**30**	**25**	**no**	**none**	**OAD—metformin**	**5.8**	**6.2**	**6.6 (48.6)**	**N/A**	**multiple generations affected**
F016	P038	no diabetes	3	N/A	N/A	none	N/A	N/A	N/A	N/A	N/A	multiple generations affected
F016	P040	15	33	N/A	N/A	none	diet	7.6	N/A	N/A	N/A	multiple generations affected
F016	P041	N/A	5	N/A	N/A	none	diet	5.8	8.1	6.1 (43.2)	N/A	multiple generations affected
F016	P042	N/A	55	N/A	N/A	N/A	N/A	N/A	N/A	N/A	N/A	multiple generations affected
F016	P044	N/A	14	N/A	N/A	N/A	N/A	N/A	N/A	N/A	N/A	multiple generations affected
**F018**	**P047**	**10**	**14**	**N/A**	**N/A**	**none**	**diet**	**6.5**	**N/A**	**6.3 (45.4)**	**N/A**	**no family members tested**
**F027**	**P063**	**N/A**	**25**	**N/A**	**N/A**	**none**	**diet**	**6.1**	**8.8**	**6.2 (44.3)**	**N/A**	**no family members tested**
**F028**	**P064**	**10**	**12**	**19.4**	**no**	**none**	**N/A**	**7.1**	**8.5**	**N/A**	**N/A**	**multiple generations affected**
F028	P065	N/A	11	23.2	no	none	OAD—metformin	6.2	7.1	6.5 (47.5)	N/A	multiple generations affected
F028	P066	N/A	41	N/A	N/A	none	none	6.7	6.7	N/A	N/A	multiple generations affected
**F031**	**P069**	**8**	**11**	**16.2**	**no**	**none**	**OAD—metformin**	**7.6**	**7.9**	**6.7 (49.7)**	**75.5**	**multiple generations affected**
F031	P070	27	40	N/A	N/A	N/A	diet	N/A	N/A	N/A	N/A	multiple generations affected
**F035**	**P074**	**8**	**11**	**19.1**	**no**	**none**	**diet**	**6.0**	**15.2**	**6.2 (43.2)**	**75.5**	**multiple generations affected**
F035	P075	26	40	23.2	no	N/A	OAD—metformin	N/A	N/A	N/A	N/A	multiple generations affected
**F038**	**P078**	**N/A**	**36**	**N/A**	**N/A**	**N/A**	**N/A**	**N/A**	**N/A**	**N/A**	**N/A**	**multiple generations affected**
F038	P079	7	12	15.2	no	none	none	6.5	8.6	N/A	N/A	multiple generations affected
**F041**	**P082**	**8**	**10**	**14.31**	**no**	**none**	**diet**	**8.1**	**11.6**	**6.2 (44.3)**	**75.5**	**de novo**
**F042**	**P085**	**4**	**7**	**15.7**	**no**	**none**	**diet**	**6.0**	**8.5**	**6.4 (46.4)**	**75.5**	**siblings positive, parents not tested**
F042	P086	1	1	N/A	N/A	N/A	N/A	N/A	N/A	4.8 (29.0)	N/A	siblings positive, parents not tested
**F043**	**P088**	**20**	**34**	**21.5**	**no**	**none**	**diet**	**7.9**	**N/A**	**6.4 (46.4)**	**75.5**	**no family members tested**
**F044**	**P089**	**14**	**17**	**24**	**no**	**none**	**insulin**	**5.1**	**N/A**	**6.8 (50.8)**	**49.4**	**multiple generations affected**
F044	P090	N/A	48	29.5	no	renal cysts	N/A	6.9	N/A	6.7 (49.7)	N/A	multiple generations affected
F044	P091	46	69	21	no	TIA, glaucoma, osteoporosis	OAD—metformin	5.1	N/A	6.6 (48.6)	N/A	multiple generations affected
**F046**	**P104**	**18**	**45**	**22.0**	**no**	**none**	**none**	**N/A**	**N/A**	**5.5 (36.6)**	**75.5**	**multiple generations affected**
F046	P105	13	18	23.7	no	none	insulin	7.3	11.2	6.7 (49.7)	49.4	multiple generations affected
F046	P106	14	24	18.5	no	none	insulin	6.0	9.5	6.2 (44.3)	75.5	multiple generations affected
F046	P108	15	20	19.4	no	none	insulin	7.7	8.7	7.0 (53.0)	8.2	multiple generations affected
F046	P113	N/A	1	N/A	N/A	N/A	N/A	N/A	N/A	N/A	N/A	multiple generations affected
F046	P738	N/A	3	15.5	no	none	none	5.7	N/A	6.0 (42.1)	N/A	multiple generations affected
F046	P739	N/A	6	15.7	no	none	none	5.8	N/A	6.1 (43.2)	N/A	multiple generations affected
**F062**	**P128**	**3**	**3**	**N/A**	**N/A**	**none**	**none**	**6.0**	**5.4**	**N/A**	**N/A**	**multiple generations affected**
F062	P129	no diabetes	1	N/A	N/A	N/A	N/A	N/A	N/A	N/A	N/A	multiple generations affected
F062	P130	no diabetes	N/A	N/A	N/A	N/A	N/A	N/A	N/A	N/A	N/A	multiple generations affected
**F065**	**P133**	**11**	**23**	**30**	**yes**	**N/A**	**insulin**	**N/A**	**N/A**	**6.6 (48.6)**	**12.6**	**no family members tested**
**F069**	**P141**	**16**	**18**	**18.7**	**no**	**none**	**OAD—metformin**	**6.8**	**N/A**	**5.7 (38.8)**	**75.5**	**multiple generations affected**
F069	P142	N/A	51	N/A	N/A	N/A	N/A	6.9	N/A	6.4 (46.4)	N/A	multiple generations affected
F069	P143	N/A	64	N/A	N/A	N/A	N/A	7.1	N/A	6.4 (46.4)	N/A	multiple generations affected
**F070**	**P144**	**N/A**	**23**	**18.7**	**no**	**ligament tear**	**OAD—metformin**	**7.1**	**N/A**	**6.0 (42.1)**	**N/A**	**no family members tested**
**F080**	**P138**	**N/A**	**8**	**N/A**	**N/A**	**N/A**	**N/A**	**5–7**	**N/A**	**N/A**	**N/A**	**no family members tested**
**F083**	**P157**	**N/A**	**24**	**N/A**	**N/A**	**PCOS**	**none**	**6.9**	**7.9**	**N/A**	**N/A**	**multiple generations affected**
F083	P158	15	50	N/A	N/A	N/A	OAD—metformin	7.78	N/A	6.3 (45.4)	N/A	multiple generations affected
**F085**	**P160**	**16**	**18**	**20.1**	**no**	**none**	**diet**	**7.1**	**7.9**	**6.8 (50.8)**	**75.5**	**multiple generations affected**
F085	P161	15	32	17.8	no	none	diet	6	N/A	6.1 (43.2)	75.5	multiple generations affected
F085	P162	2	2	N/A	N/A	granuloma annulare	diet	6.1	N/A	6.1 (43.2)	N/A	multiple generations affected
**F092**	**P170**	**N/A**	**15**	**N/A**	**N/A**	**none**	**diet**	**6.6**	**9.4**	**6.5 (47.5)**	**N/A**	**no family members tested**
**F094**	**P172**	**10**	**15**	**19.4**	**no**	**none**	**diet**	**7.2**	**9.6**	**6.4 (46.4)**	**75.5**	**parents not tested**
**F101**	**P192**	**6**	**7**	**13.4**	**no**	**none**	**none**	**6.0**	**10.0**	**6.0 (42.1)**	**75.5**	**multiple generations affected**
F101	P193	no diabetes	46	normal	no	N/A	N/A	N/A	N/A	N/A	N/A	multiple generations affected
**F103**	**P196**	**5**	**6**	**17**	**no**	**none**	**diet**	**6.2**	**5.8**	**6.3 (45.4)**	**75.5**	**multiple generations affected**
F103	P197	36	42	24	no	none	insulin	6.9	13.6	6.0 (42.1)	N/A	multiple generations affected
**F105**	**P199**	**9**	**14**	**20.9**	**no**	**N/A**	**diet**	**7.3**	**N/A**	**N/A**	**N/A**	**multiple generations affected**
F105	P200	25	37	30.1	yes	none	none	N/A	N/A	N/A	N/A	multiple generations affected
F105	P202	N/A	6	15.1	no	none	none	N/A	N/A	N/A	N/A	multiple generations affected
**F107**	**P204**	**16**	**17**	**19**	**no**	**none**	**diet**	**7.9**	**8.8**	**7.0 (53.0)**	**75.5**	**no family members tested**
**F108**	**P205**	**21**	**30**	**15.1**	**no**	**none**	**insulin**	**6.2**	**10.4**	**6.0 (42.1)**	**75.5**	**no family members tested**
**F113**	**P210**	**12**	**17**	**17**	**no**	**none**	**insulin**	**N/A**	**N/A**	**6.3 (45.4)**	**12.6**	**multiple generations affected**
F113	P211	43	45	22.2	no	none	diet	6.9	9.2	6.3 (45.4)	N/A	multiple generations affected
**F116**	**P214**	**7**	**19**	**19.8**	**no**	**headache, elevated RR**	**OAD—metformin**	**5.8**	**14**	**6.8 (50.8)**	**75.5**	**cousin positive, parents not tested**
F116	P215	9	15	21.1	no	none	diet	5.8	6.4	6.5 (47.5)	75.5	cousin positive, parents not tested
**F122**	**P222**	**14**	**20**	**25**	**no**	**N/A**	**diet**	**6.5**	**N/A**	**6.2 (44.3)**	**75.5**	**siblings positive, parents not tested**
F122	P223	14	24	24	no	none	diet	6.3	N/A	N/A	N/A	siblings positive, parents not tested
**F126**	**P227**	**14**	**36**	**22.5**	**no**	**PCOS**	**OAD—metformin**	**5.9**	**>11**	**5.8 (39.9)**	**75.5**	**no family members tested**
**F131**	**P232**	**19**	**28**	**19.4**	**no**	**none**	**none**	**8.4**	**N/A**	**6.8 (50.8)**	**75.5**	**no family members tested**
**F133**	**P236**	**13**	**14**	**19**	**no**	**none**	**diet**	**6.4**	**9.4**	**6.4 (46.4)**	**75.5**	**multiple generations affected**
F133	P237	11	18	19	no	none	insulin	N/A	N/A	6.4 (46.4)	8.2	multiple generations affected
F133	P240	31	42	21	no	none	OAD	7.8	10.00	6.4 (46.4)	58	multiple generations affected
F133	P241	57	63	38	yes	IHD	OAD	8.7	10	N/A	N/A	multiple generations affected
**F145**	**P257**	**6**	**12**	**15.2**	**no**	**none**	**insulin**	**N/A**	**N/A**	**7.1 (54.1)**	**12.6**	**multiple generations affected**
F145	P258	37	38	N/A	N/A	N/A	N/A	7.3	7.8	5.8 (39.9)	N/A	multiple generations affected
F145	P259	no diabetes	2016	N/A	N/A	none	N/A	7.0	5.9	N/A	N/A	multiple generations affected
F145	P260	4	5	N/A	N/A	none	none	N/A	9.7	6.8 (50.8)	N/A	multiple generations affected
F145	P261	39	40	24.3	no	N/A	N/A	7.2	5.9	6.3 (45.4)	N/A	multiple generations affected
**F150**	**P266**	**35**	**59**	**21.6**	**no**	**none**	**OAD—metformin**	**10.1**	**N/A**	**7.4 (57.4)**	**15.1**	**parents not tested**
**F153**	**P271**	**9**	**16**	**23.6**	**no**	**none**	**diet**	**6.9**	**12.0**	**6.2 (44.3)**	**75.5**	**no family members tested**
**F162**	**P280**	**9**	**12**	**32.4**	**yes**	**acanthosis nigricans**	**OAD—metformin**	**N/A**	**N/A**	**6.5 (47.5)**	**75.5**	**multiple generations affected**
F162	P281	3	4	14.1	no	N/A	diet	4.7	5.7	6.4 (46.4)	75.5	multiple generations affected
F162	P282	N/A	2	N/A	N/A	N/A	N/A	N/A	N/A	N/A	N/A	multiple generations affected
F162	P283	no diabetes	21	N/A	N/A	N/A	none	5.4	6.2	N/A	N/A	multiple generations affected
**F163**	**P284**	**7**	**11**	**29.0**	**yes**	**none**	**none**	**10.0**	**6.3**	**6.2 (44.3)**	**75.5**	**multiple generations affected**
F163	P285	9	13	23.5	no	none	none	6.6	10.9	5.9 (41.0)	75.5	multiple generations affected
F163	P286	N/A	43	N/A	N/A	N/A	N/A	N/A	N/A	N/A	N/A	multiple generations affected
**F165**	**P288**	**10**	**11**	**18.0**	**no**	**N/A**	**diet**	**7**	**10.8**	**5.9 (41.0)**	**75.5**	**no family members tested**
**F167**	**P290**	**13**	**13**	**19.2**	**no**	**none**	**diet**	**5.2**	**10.7**	**5.7 (38.8)**	**75.5**	**multiple generations affected**
F167	P292	no diabetes	48	N/A	N/A	N/A	N/A	N/A	N/A	N/A	N/A	multiple generations affected
**F173**	**P298**	**1**	**2**	**15**	**no**	**N/A**	**diet**	**5.6**	**9.9**	**6.4 (46.4)**	**75.5**	**multiple generations affected**
F173	P299	16	33	N/A	N/A	N/A	insulin	N/A	N/A	N/A	N/A	multiple generations affected
**F187**	**P319**	**1**	**4**	**16.5**	**no**	**none**	**none**	**6.3—7**	**5.8—7**	**6.5 (47.5)**	**75.5**	**no family members tested**
**F191**	**P323**	**1**	**12**	**16.8**	**no**	**none**	**diet**	**6.6**	**7.3**	**6.6 (48.6)**	**75.5**	**no family members tested**
**F197**	**P335**	**13**	**27**	**21.9**	**no**	**none**	**diet**	**6.9**	**8.5**	**6.5 (47.5)**	**75.5**	**no family members tested**
**F200**	**P338**	**5**	**18**	**17.7**	**no**	**none**	**diet**	**6.17**	**8.3**	**6.8 (50.8)**	**75.5**	**multiple generations affected**
F200	P339	47	51	23.9	no	none	OAD—metformin + sulphonylurea	N/A	N/A	N/A	N/A	multiple generations affected
**F201**	**P340**	**23**	**31**	**19.7**	**no**	**none**	**OAD—** **sulphonylurea**	**6.6**	**11.2**	**6.1 (43.2)**	**75.5**	**multiple generations affected**
F201	P341	57	60	30.8	yes	N/A	diet	6.9	14.5	6.3 (45.4)	N/A	multiple generations affected
F201	P342	22	35	23.1	no	none	diet	7.3	9.8	5.8 (39.9)	75.5	multiple generations affected
**F202**	**P343**	**5**	**6**	**17.0**	**no**	**none**	**none**	**6.2**	**8.2**	**6.4 (46.4)**	**75.5**	**siblings positive, parents not tested**
F202	P344	9	10	15.4	no	none	none	6.5	6.9	6.9 (51.9)	75.5	siblings positive, parents not tested
**F205**	**P349**	**4**	**10**	**16.5**	**no**	**none**	**diet**	**5.8**	**8.5**	**5.7 (38.8)**	**75.5**	**no family members tested**
**F209**	**P353**	**9**	**43**	**29**	**no**	**none**	**OAD—metformin**	**7.2**	**9.5**	**7.0 (53.0)**	**75.5**	**no family members tested**
**F213**	**P357**	**N/A**	**12**	**17.8**	**no**	**none**	**diet**	**7.1**	**8.6**	**6.2 (44.3)**	**N/A**	**multiple generations affected**
F213	P358	N/A	34	N/A	N/A	N/A	diet	N/A	N/A	N/A	N/A	multiple generations affected
**F216**	**P361**	**N/A**	**14**	**16.2**	**no**	**none**	**diet**	**5.6**	**5.9**	**6.3 (45.4)**	**N/A**	**no family members tested**
**F219**	**P364**	**32**	**39**	**25.2**	**no**	**none**	**diet**	**6.9**	**8.7**	**5.5 (36.6)**	**62.4**	**parents not tested**
**F227**	**P373**	**7**	**21**	**25.7**	**no**	**none**	**insulin**	**7.0**	**14.7**	**6.1 (43.2)**	**75.5**	**multiple generations affected**
F227	P374	N/A	15	15.5	no	N/A	N/A	6.8	N/A	N/A	N/A	multiple generations affected
F227	P377	45	51	33	yes	none	OAD—metformin	7.8	8.9	N/A	N/A	multiple generations affected
**F244**	**P394**	**15**	**16**	**17.6**	**no**	**none**	**diet**	**6.5**	**8.8**	**6.2 (44.3)**	**75.5**	**no family members tested**
**F246**	**P396**	**6**	**7**	**19.9**	**yes**	**none**	**none**	**6.7**	**9.4**	**6.3 (45.4)**	**75.5**	**multiple generations affected**
F246	P398	no diabetes	43	N/A	N/A	N/A	N/A	7.5	N/A	7.0 (53.0)	N/A	multiple generations affected
**F250**	**P403**	**N/A**	**35**	**N/A**	**N/A**	**cardiac**	**N/A**	**N/A**	**N/A**	**N/A**	**N/A**	**no family members tested**
**F263**	**P426**	**18**	**31**	**21.5**	**no**	**N/A**	**OAD—metformin**	**6.9**	**8.5**	**6.4 (46.4)**	**75.5**	**no family members tested**
**F272**	**P435**	**20**	**22**	**17.4**	**no**	**none**	**none**	**6.7**	**22.9**	**6.0 (42.1)**	**75.5**	**no family members tested**
**F273**	**P436**	**N/A**	**18**	**19.5**	**no**	**N/A**	**N/A**	**5.8**	**N/A**	**N/A**	**N/A**	**no family members tested**
**F274**	**P437**	**5**	**16**	**32.7**	**yes**	**hypertension, obesity**	**OAD—metformin**	**6.5**	**9.4**	**6.5 (47.5)**	**75.5**	**siblings positive, parents not tested**
F274	P438	2	7	16.7	no	none	diet	6.7	N/A	6.4 (46.4)	75.5	siblings positive, parents not tested
**F275**	**P439**	**8**	**10**	**16.3**	**no**	**none**	**diet**	**6.1**	**8.3**	**6.1 (43.2)**	**75.5**	**no family members tested**
**F280**	**P444**	**N/A**	**10**	**N/A**	**N/A**	**N/A**	**N/A**	**N/A**	**N/A**	**N/A**	**N/A**	**no family members tested**
**F291**	**P455**	**10**	**16**	**19.2**	**no**	**none**	**insulin**	**6.5**	**11.7**	**6.8 (50.8)**	**4**	**no family members tested**
**F296**	**P460**	**13**	**18**	**N/A**	**N/A**	**none**	**insulin**	**N/A**	**N/A**	**N/A**	**N/A**	**no family members tested**
**F306**	**P470**	**7**	**8**	**14.6**	**no**	**repeated acute laryngitis**	**none**	**6.10**	**N/A**	**6.0 (42.0)**	**75.5**	**no family members tested**
**F310**	**P477**	**14**	**15**	**22.2**	**no**	**N/A**	**N/A**	**N/A**	**N/A**	**6.4 (46.4)**	**75.5**	**mother no carrier, father not tested**
**F314**	**P482**	**4**	**6**	**14.9**	**no**	**none**	**diet**	**N/A**	**N/A**	**6.0 (42.1)**	**75.5**	**no family members tested**
**F316**	**P488**	**30**	**37**	**20.6**	**no**	**none**	**OAD—metformin**	**6.3**	**6.9**	**6.3 (45.4)**	**35.8**	**no family members tested**
**F349**	**P532**	**17**	**39**	**20.6**	**no**	**none**	**OAD—metformin**	**5.7**	**N/A**	**6.4 (46.4)**	**75.5**	**no family members tested**
**F353**	**P536**	**14**	**23**	**26.7**	**no**	**none**	**diet**	**6.9**	**9.6**	**6.5 (47.5)**	**75.5**	**no family members tested**
**F373**	**P557**	**8**	**18**	**19.2**	**no**	**N/A**	**diet**	**5.7**	**9**	**5.6 (37.7)**	**75.5**	**no family members tested**
**F374**	**P558**	**2**	**19**	**20**	**no**	**N/A**	**diet**	**6.0**	**9.4**	**6.7 (49.7)**	**75.5**	**no family members tested**
**F375**	**P559**	**8**	**10**	**normal**	**no**	**none**	**diet**	**7.9**	**9.5**	**6.7 (49.7)**	**N/A**	**de novo**
**F381**	**P570**	**12**	**12**	**21.2**	**no**	**none**	**diet**	**5.3**	**8.8**	**6.8 (50.8)**	**75.5**	**multiple generations affected**
F381	P572	2	41	24	no	N/A	diet	N/A	N/A	N/A	N/A	multiple generations affected
**F382**	**P574**	**13**	**13**	**22**	**no**	**none**	**diet**	**7.7**	**9.8**	**6.3 (45.4)**	**75.5**	**multiple generations affected**
F382	P575	N/A	41	N/A	N/A	N/A	N/A	N/A	N/A	N/A	N/A	multiple generations affected
F382	P577	N/A	61	N/A	N/A	N/A	N/A	N/A	N/A	N/A	N/A	multiple generations affected
F382	P578	N/A	29	N/A	N/A	N/A	N/A	N/A	N/A	N/A	N/A	multiple generations affected
**F389**	**P586**	**28**	**31**	**20**	**no**	**none**	**diet**	**7.2**	**9.5**	**6.3 (45.4)**	**62.4**	**no family members tested**
**F400**	**P598**	**14**	**18**	**21**	**no**	**none**	**none**	**6.7**	**8.9**	**5.7 (38.8)**	**75.5**	**no family members tested**
**F403**	**P601**	**10**	**11**	**15.1**	**no**	**none**	**insulin**	**6.0**	**11.0**	**6.3 (45.4)**	**12.6**	**mother no carrier, father not tested**
F403	P604	no diabetes	7	N/A	N/A	N/A	N/A	N/A	N/A	N/A	N/A	mother no carrier, father not tested
**F408**	**P610**	**5**	**18**	**18.7**	**no**	**none**	**insulin**	**7.3**	**N/A**	**6.4 (46.4)**	**1.9**	**siblings positive, parents not tested**
F408	P611	14	14	17.9	no	none	diet	7.2	8.8	7.1 (54.1)	75.5	siblings positive, parents not tested
**F411**	**P614**	**12**	**12**	**14.7**	**no**	**none**	**diet**	**5.9**	**9.3**	**7.0 (53.0)**	**75.5**	**multiple generations affected**
F411	P616	no diabetes	11	N/A	N/A	N/A	N/A	N/A	N/A	N/A	N/A	multiple generations affected
F411	P617	no diabetes	42	26	no	none	diet	7.1	9.1	6.2 (44.3)	N/A	multiple generations affected
**F433**	**P639**	**N/A**	**13**	**N/A**	**N/A**	**N/A**	**N/A**	**N/A**	**N/A**	**N/A**	**N/A**	**no family members tested**
**F434**	**P640**	**N/A**	**10**	**N/A**	**N/A**	**N/A**	**N/A**	**N/A**	**N/A**	**N/A**	**N/A**	**no family members tested**
**F435**	**P641**	**7**	**13**	**18**	**no**	**none**	**insulin**	**N/A**	**N/A**	**7.1 (54.1)**	**8.2**	**multiple generations affected**
F435	P642	24	37	24	no	none	none	N/A	N/A	7.2 (55.2)	62.4	multiple generations affected
**F454**	**P665**	**10**	**11**	**17.4**	**no**	**possible coeliac disease**	**none**	**6.2**	**5.9**	**N/A**	**N/A**	**no family members tested**
**F455**	**P666**	**3**	**4**	**N/A**	**N/A**	**N/A**	**diet**	**4.9**	**12.7**	**6.1 (43.2)**	**N/A**	**multiple generations affected**
F455	P667	N/A	N/A	N/A	N/A	N/A	N/A	N/A	N/A	N/A	N/A	multiple generations affected
**F463**	**P675**	**20**	**27**	**18.9**	**no**	**none**	**OAD—metformin + sulphonylurea**	**7.2**	**N/A**	**6.6 (48.6)**	**75.5**	**no family members tested**
**F472**	**P684**	**1**	**2**	**N/A**	**N/A**	**N/A**	**N/A**	**6.4**	**8.6**	**6.9 (51.9)**	**N/A**	**multiple generations affected**
F472	P733	N/A	50	N/A	N/A	N/A	N/A	N/A	N/A	N/A	N/A	multiple generations affected
**F482**	**P696**	**6**	**6**	**28**	**yes**	**N/A**	**diet**	**6.4**	**11.0**	**6.2 (44.3)**	**75.5**	**no family members tested**
**F499**	**P713**	**N/A**	**21**	**N/A**	**N/A**	**N/A**	**N/A**	**N/A**	**N/A**	**N/A**	**N/A**	**no family members tested**

Index patients are shown in bold. Age at diabetes: N/A—the patient has diabetes, but no information was received regarding the age of diagnosis; no diabetes—no information was given in the application form that the patient shows any signs of diabetes. dg—diagnosis; OAD—oral antidiabetic drug; IHD—Ischemic Heart Disease; PAD—Peripheral Arterial Disease; TIA—transient ischemic attack; PCOS—Polycystic Ovary Syndrome; RR—respiratory rate. * BMI data refers to the time of referral for genetic testing.

**Table 5:** The distribution of the types of therapy received before proper genetic diagnosis.

Therapy	Number of Patients
insulin	19 (11.7%)
OAD—sulphonylurea	2 (1.2%)
OAD—metformin	19 (11.7%)
OAD—metformin and sulphonylurea	2 (1.2%)
OAD—acarbose	1 (0.6%)
other OAD	2 (1.2%)
diet	56 (34.4%)
no treatment	24 (14.7%)
N/A	38 (23.3%)

## Data Availability

The data presented in this study are available on request from the corresponding author.

## Supplementary Materials

The following are available online at https://www.mdpi.com/article/10.3390/life11080771/s1, Table S1. The list of genes examined with the different library preparation kits; Table S2. Methods used for testing the index patients; Table S3. Clinical data of patients with HNF1A mutation; Table S4. Clinical data of patients having a mutation in other MODY-causing genes.
