# Supplementary material for: A Comprehensive Analysis of Hungarian MODY Patients—Part II: Glucokinase MODY Is the Most Prevalent Subtype Responsible for about 70% of Confirmed Cases
Source: Life (Basel). 2021 Jul 30;11(8):771. doi: 10.3390/life11080771 (PMC8400228; doi:10.3390/life11080771)
Supplement: Supplementary file 1 [file life-11-00771-s001.zip › life-1298213-supplementary.pdf]

life

Supplementary Materials for A Comprehensive Analysis of Hungarian MODY Patients–Part I: Gene Panel Sequencing Reveals Pathogenic Mutations in *HNF1A, HNF1B, HNF4A, ABCC8* and *INS* Genes

Table S1. The list of genes examined with the different library preparation kits.

MODY MASTR kit (Multiplicom, Niel, Belgium)	7 genes: *ABCC8, GCK, HNF1A, HNF1B, HNF4A, INS, KCNJ11*
custom DNA library preparation kit (Qiagen, GmbH, Hilden, Germany)	17 genes: *ABCC8, GCK, HNF1A, HNF1B, HNF4A, INS, KCNJ11, SLC16A1, GLUD1, PDX1, INSR, KLF11, NEUROD1, APPL1, HADH, PAX4, BLK*
custom-designed gene panel (Twist Bioscience, South San Francisco, CA, USA)	18 genes: *ABCC8, GCK, HNF1A, HNF1B, HNF4A, INS, KCNJ11, SLC16A1, GLUD1, PDX1, INSR, KLF11, NEUROD1, APPL1, HADH, PAX4, BLK, RFX6*<br>20 genes: *ABCC8, GCK, HNF1A, HNF1B, HNF4A, INS, KCNJ11, SLC16A1, GLUD1, PDX1, INSR, KLF11, NEUROD1, APPL1, HADH, PAX4, BLK, RFX6, CEL, WFS1*

Table S2. Methods used for testing the index patients.

Sample ID	Result	Method
P001	Negative	Sanger: *HNF1A*; Sanger: *HNF4A*
P002	Positive	Sanger: *GCK*
P003	Negative	Sanger: *HNF1A*
P004	Negative	Sanger: *HNF1A*
P015	Positive	Sanger: *GCK*
P020	Positive	Sanger: *HNF1A*
P024	Negative	Sanger: *HNF1A*
P025	Negative	Sanger: *HNF1A*
P028	Positive	Sanger: *GCK*
P032	Positive	Sanger: *HNF1A*
P034	Negative	Sanger: *HNF1A*
P035	Negative	Sanger: *HNF1A*
P036	Negative	Sanger: *HNF1A*
P037	Positive	Sanger: *GCK*
P046	Negative	Sanger: *HNF1A*
P047	Positive	Sanger: *GCK*
P049	Positive	Sanger: *HNF1A*
P056	Negative	Sanger: *GCK*
P057	Negative	Sanger: *GCK*
P058	Positive	Sanger: *GCK*; Sanger: *HNF1A*
P062	Negative	Sanger: *HNF1A*
P063	Positive	Sanger: *GCK*
P064	Positive	Sanger: *GCK*
P068	Negative	Sanger: *GCK*
P069	Positive	Sanger: *GCK*
P071	Negative	Sanger: *GCK*; Sanger: *HNF1A*
P072	Negative	Sanger: *HNF1A*
P073	Negative	Sanger: *HNF1A*

P074	Positive	Sanger: *GCK*; Sanger: *HNF1A*
P076	Negative	Sanger: *HNF1A*; Sanger: *HNF4A*
P077	Negative	Sanger: *GCK*
P078	Positive	Sanger: *GCK*
P080	Negative	Sanger: *HNF1A*
P081	Negative	Sanger: *HNF1A*
P082	Positive	Sanger: *GCK*
P085	Positive	Sanger: *GCK*
P088	Positive	Sanger: *GCK*
P089	Positive	Sanger: *GCK*
P101	Negative	Sanger: *GCK*
P104	Positive	Sanger: *GCK*
P095	Negative	NGS: TWIST (20 genes)
P099	Negative	NGS: TWIST (20 genes)
P119	Negative	Sanger: *GCK*; Sanger: *HNF1A*
P120	Negative	Sanger: *GCK*; Sanger: *HNF1A*
P110	Negative	NGS: TWIST (20 genes)
P112	Negative	NGS: TWIST (20 genes)
P125	Negative	Sanger: *HNF1A*
P126	Negative	Sanger: *GCK*
P127	Negative	Sanger: *GCK*
P128	Positive	Sanger: *GCK*
P131	Negative	Sanger: *HNF1A*; Sanger: *HNF4A*
P132	Negative	Sanger: *HNF1A*
P133	Positive	Sanger: *GCK*
P140	Negative	Sanger: *HNF1A*
P141	Positive	Sanger: *GCK*
P144	Positive	NGS: Multiplicom (7 genes)
P145	Negative	Sanger: *GCK*; Sanger: *HNF1A*
P146	Negative	Sanger: *GCK*
P147	Negative	Sanger: *GCK*
P149	Negative	MLPA
P150	Positive	MLPA
P138	Positive	NGS: TWIST (20 genes)
P154	Negative	Sanger: *GCK*
P155	Negative	Sanger: *HNF1A*
P157	Positive	Sanger: *GCK*
P159	Negative	Sanger: *GCK*
P160	Positive	Sanger: *GCK*; Sanger: *HNF1A*
P163	Negative	NGS: Multiplicom (7 genes)
P164	Negative	Sanger: *HNF1A*; Sanger: *HNF4A*; MLPA
P166	Negative	MLPA
P168	Positive	Sanger: *HNF4A*; MLPA
P170	Positive	Sanger: *GCK*
P171	Negative	Sanger: *HNF1A*; Sanger: *HNF4A*; MLPA
P172	Positive	Sanger: *GCK*
P174	Positive	Sanger: *HNF1A*
P177	Positive	Sanger: *GCK*; Sanger: *HNF1A*
P178	Positive	Sanger: *GCK*; Sanger: *HNF1A*; Sanger: *HNF4A*; MLPA

P186	Positive	Sanger: *HNF1A*
P191	Negative	Sanger: *HNF1A*
P192	Positive	Sanger: *GCK*
P195	Negative	Sanger: *HNF1A*; Sanger: *HNF4A*; MLPA
P196	Positive	Sanger: *GCK*; Sanger: *HNF1A*
P198	Positive	Sanger: *HNF1A*
P199	Positive	Sanger: *GCK*
P203	Negative	Sanger: *GCK*
P204	Positive	Sanger: *GCK*
P205	Positive	Sanger: *GCK*; Sanger: *HNF1A*; Sanger: *HNF4A*
P207	Negative	Sanger: *GCK*
P208	Negative	Sanger: *GCK*
P209	Negative	Sanger: *HNF1A*; Sanger: *HNF4A*; MLPA
P210	Positive	Sanger: *GCK*
P213	Negative	NGS: Qiagen (17 genes)
P214	Positive	Sanger: *GCK*; Sanger: *HNF1A*; Sanger: *HNF4A*
P216	Negative	NGS: Junior
P218	Negative	Sanger: *GCK*; Sanger: *HNF1A*
P219	Negative	Sanger: *GCK*; Sanger: *HNF1A*
P220	Negative	Sanger: *GCK*; Sanger: *HNF1A*
P221	Negative	NGS: Multiplicom (7 genes); MLPA
P222	Positive	Sanger: *GCK*
P224	Negative	Sanger: *GCK*; Sanger: *HNF1A*; Sanger: *HNF4A*; MLPA
P225	Negative	Sanger: *GCK*; Sanger: *HNF1A*; Sanger: *HNF4A*
P227	Positive	NGS: Multiplicom (7 genes); MLPA
P228	Negative	Sanger: *GCK*
P229	Positive	Sanger: *HNF1A*
P230	Negative	NGS: Junior; MLPA
P231	Negative	Sanger: *HNF1A*; Sanger: *HNF4A*
P232	Positive	Sanger: *GCK*
P236	Positive	Sanger: *GCK*
P243	Negative	Sanger: *GCK*; Sanger: *HNF1A*; Sanger: *HNF4A*; Sanger: *KCNJ11*; MLPA
P244	Negative	Sanger: *GCK*; MLPA
P245	Negative	Sanger: *GCK*
P246	Negative	Sanger: *HNF1A*; Sanger: *HNF4A*
P247	Negative	NGS: Junior
P248	Negative	Sanger: *GCK*; Sanger: *HNF1A*
P249	Negative	Sanger: *GCK*
P250	Positive	NGS: Junior
P254	Negative	Sanger: *GCK*; Sanger: *HNF1A*; MLPA
P255	Negative	Sanger: *GCK*; Sanger: *HNF1A*
P256	Negative	NGS: Junior
P257	Positive	NGS: Junior
P262	Negative	NGS: Junior
P263	Negative	NGS: Junior
P264	Negative	NGS: Junior
P265	Negative	NGS: Junior
P266	Positive	NGS: Junior
P269	Negative	NGS: Junior

P271	Positive	NGS: Junior
P272	Negative	NGS: Junior
P273	Positive	NGS: Junior
P274	Negative	NGS: Junior
P275	Negative	NGS: Junior
P276	Negative	NGS: Junior
P277	Positive	NGS: Junior
P278	Negative	NGS: Junior
P279	Negative	NGS: Junior
P280	Positive	NGS: Junior
P285	Positive	NGS: Junior
P287	Negative	MLPA
P288	Positive	NGS: Junior
P289	Negative	NGS: Junior
P290	Positive	NGS: Junior
P294	Negative	NGS: Junior; MLPA
P295	Negative	NGS: Junior
P296	Negative	NGS: Junior
P297	Positive	NGS: Junior; MLPA
P298	Positive	Sanger: *GCK*
P300	Negative	NGS: Junior
P301	Negative	NGS: Junior; MLPA
P316	Negative	NGS: Junior
P317	Negative	NGS: Multiplicom (7 genes)
P318	Negative	NGS: Multiplicom (7 genes)
P319	Positive	NGS: Multiplicom (7 genes)
P320	Negative	NGS: Multiplicom (7 genes)
P321	Negative	NGS: Qiagen (17 genes)
P322	Negative	NGS: Multiplicom (7 genes)
P323	Positive	NGS: Multiplicom (7 genes)
P324	Positive	NGS: Multiplicom (7 genes)
P325	Negative	NGS: Qiagen (17 genes)
P329	Negative	NGS: Multiplicom (7 genes)
P330	Negative	NGS: Multiplicom (7 genes)
P331	Positive	NGS: Multiplicom (7 genes)
P335	Positive	NGS: Multiplicom (7 genes)
P336	Negative	NGS: Multiplicom (7 genes)
P337	Negative	NGS: Multiplicom (7 genes)
P338	Positive	NGS: Multiplicom (7 genes)
P340	Positive	NGS: Multiplicom (7 genes)
P343	Positive	NGS: Multiplicom (7 genes)
P345	Negative	NGS: Multiplicom (7 genes)
P346	Positive	NGS: Multiplicom (7 genes)
P349	Positive	NGS: Multiplicom (7 genes)
P350	Negative	NGS: Multiplicom (7 genes)
P351	Negative	NGS: Multiplicom (7 genes)
P352	Negative	NGS: Multiplicom (7 genes)
P353	Positive	NGS: Multiplicom (7 genes)
P354	Negative	NGS: Multiplicom (7 genes)

P355	Negative	NGS: Multiplicom (7 genes)
P356	Negative	NGS: Multiplicom (7 genes)
P357	Positive	NGS: Multiplicom (7 genes)
P359	Negative	NGS: Multiplicom (7 genes)
P360	Negative	NGS: Multiplicom (7 genes)
P361	Positive	NGS: Multiplicom (7 genes)
P362	Negative	NGS: Multiplicom (7 genes)
P363	Negative	NGS: Multiplicom (7 genes); MLPA
P364	Positive	NGS: Multiplicom (7 genes)
P366	Negative	NGS: Multiplicom (7 genes)
P367	Negative	NGS: Qiagen (17 genes)
P368	Negative	NGS: Multiplicom (7 genes)
P371	Negative	NGS: Multiplicom (7 genes)
P372	Negative	NGS: Multiplicom (7 genes)
P373	Positive	NGS: Multiplicom (7 genes)
P378	Positive	NGS: Multiplicom (7 genes)
P379	Negative	NGS: Multiplicom (7 genes)
P380	Negative	NGS: Multiplicom (7 genes)
P381	Negative	NGS: Multiplicom (7 genes)
P382	Negative	NGS: Multiplicom (7 genes)
P384	Positive	NGS: Multiplicom (7 genes)
P387	Negative	NGS: Multiplicom (7 genes)
P388	Negative	NGS: Multiplicom (7 genes)
P389	Negative	NGS: TWIST (18 genes); MLPA
P390	Negative	NGS: Multiplicom (7 genes)
P391	Negative	NGS: Multiplicom (7 genes)
P394	Positive	NGS: Multiplicom (7 genes)
P395	Negative	NGS: Multiplicom (7 genes)
P396	Positive	NGS: Multiplicom (7 genes)
P401	Negative	NGS: Multiplicom (7 genes)
P403	Positive	NGS: Multiplicom (7 genes)
P404	Negative	NGS: Multiplicom (7 genes)
P405	Negative	NGS: Multiplicom (7 genes)
P406	Negative	NGS: Multiplicom (7 genes)
P413	Negative	NGS: Multiplicom (7 genes); MLPA
P414	Negative	NGS: Multiplicom (7 genes)
P418	Positive	NGS: Multiplicom (7 genes)
P425	Negative	NGS: Multiplicom (7 genes)
P426	Positive	NGS: Multiplicom (7 genes)
P427	Negative	NGS: Multiplicom (7 genes)
P428	Negative	NGS: Multiplicom (7 genes)
P429	Negative	NGS: Multiplicom (7 genes)
P430	Negative	NGS: Multiplicom (7 genes)
P431	Negative	NGS: Multiplicom (7 genes); MLPA
P432	Negative	NGS: Multiplicom (7 genes)
P433	Negative	NGS: Qiagen (17 genes)
P434	Negative	NGS: Qiagen (17 genes)
P435	Positive	NGS: Qiagen (17 genes)
P436	Positive	NGS: Qiagen (17 genes); MLPA

P437	Positive	NGS: Qiagen (17 genes)
P439	Positive	NGS: Qiagen (17 genes)
P440	Negative	NGS: Qiagen (17 genes)
P441	Negative	NGS: Qiagen (17 genes)
P442	Negative	NGS: Qiagen (17 genes)
P443	Negative	NGS: Qiagen (17 genes)
P444	Positive	NGS: Qiagen (17 genes)
P445	Negative	NGS: Qiagen (17 genes)
P446	Negative	NGS: Qiagen (17 genes)
P447	Positive	NGS: Qiagen (17 genes)
P448	Positive	NGS: Qiagen (17 genes)
P449	Negative	NGS: Qiagen (17 genes)
P450	Negative	NGS: Qiagen (17 genes)
P451	Positive	NGS: Qiagen (17 genes)
P452	Negative	NGS: Qiagen (17 genes)
P453	Negative	NGS: Qiagen (17 genes)
P454	Negative	NGS: Qiagen (17 genes)
P455	Positive	NGS: Qiagen (17 genes)
P456	Negative	NGS: Qiagen (17 genes)
P457	Negative	NGS: Qiagen (17 genes)
P458	Negative	NGS: Qiagen (17 genes)
P459	Negative	NGS: Qiagen (17 genes)
P460	Positive	NGS: Qiagen (17 genes)
P461	Positive	NGS: Qiagen (17 genes)
P462	Negative	NGS: Qiagen (17 genes)
P463	Negative	NGS: Qiagen (17 genes)
P464	Negative	NGS: Qiagen (17 genes)
P465	Negative	NGS: Qiagen (17 genes)
P466	Positive	NGS: Qiagen (17 genes)
P467	Negative	NGS: Qiagen (17 genes)
P468	Negative	NGS: Qiagen (17 genes); MLPA
P469	Positive	NGS: Qiagen (17 genes)
P470	Positive	NGS: Qiagen (17 genes)
P471	Negative	NGS: Qiagen (17 genes)
P472	Negative	NGS: Qiagen (17 genes)
P473	Negative	NGS: Qiagen (17 genes)
P477	Positive	NGS: Qiagen (17 genes); MLPA
P479	Negative	NGS: Qiagen (17 genes)
P480	Negative	NGS: Qiagen (17 genes)
P481	Negative	NGS: Qiagen (17 genes); MLPA
P482	Positive	NGS: Qiagen (17 genes)
P483	Positive	NGS: Qiagen (17 genes)
P488	Positive	NGS: Qiagen (17 genes)
P489	Negative	NGS: Qiagen (17 genes)
P491	Negative	NGS: Qiagen (17 genes)
P492	Negative	NGS: Qiagen (17 genes)
P493	Negative	NGS: Qiagen (17 genes)
P494	Negative	NGS: Qiagen (17 genes)
P495	Negative	NGS: Qiagen (17 genes)

P496	Negative	NGS: Qiagen (17 genes)
P497	Negative	NGS: Qiagen (17 genes)
P498	Negative	NGS: Qiagen (17 genes)
P499	Negative	NGS: Qiagen (17 genes)
P500	Negative	NGS: Qiagen (17 genes)
P507	Negative	NGS: Qiagen (17 genes)
P508	Negative	NGS: Qiagen (17 genes)
P510	Negative	NGS: Qiagen (17 genes)
P511	Negative	NGS: Qiagen (17 genes)
P512	Positive	NGS: Qiagen (17 genes)
P515	Negative	NGS: Qiagen (17 genes)
P516	Negative	NGS: Qiagen (17 genes)
P517	Negative	NGS: Qiagen (17 genes)
P518	Negative	NGS: Qiagen (17 genes)
P519	Negative	NGS: Qiagen (17 genes)
P520	Negative	NGS: Qiagen (17 genes)
P521	Negative	NGS: Qiagen (17 genes)
P522	Negative	NGS: Qiagen (17 genes)
P523	Negative	NGS: Qiagen (17 genes); MLPA
P524	Negative	NGS: Qiagen (17 genes)
P528	Negative	NGS: Qiagen (17 genes)
P529	Negative	NGS: Qiagen (17 genes)
P530	Negative	NGS: Qiagen (17 genes)
P532	Positive	NGS: Multiplicom (7 genes)
P533	Negative	NGS: Qiagen (17 genes)
P534	Negative	NGS: Qiagen (17 genes)
P535	Negative	NGS: Qiagen (17 genes)
P536	Positive	NGS: Qiagen (17 genes); MLPA
P537	Negative	NGS: Qiagen (17 genes)
P538	Negative	NGS: Qiagen (17 genes)
P539	Negative	NGS: Qiagen (17 genes)
P540	Negative	NGS: Qiagen (17 genes)
P541	Negative	NGS: Qiagen (17 genes)
P542	Negative	NGS: Qiagen (17 genes)
P544	Negative	NGS: Qiagen (17 genes)
P545	Positive	NGS: Qiagen (17 genes)
P546	Negative	NGS: Qiagen (17 genes)
P547	Negative	NGS: Qiagen (17 genes)
P548	Negative	NGS: Qiagen (17 genes)
P549	Negative	NGS: Qiagen (17 genes)
P550	Positive	NGS: Qiagen (17 genes)
P551	Negative	NGS: Qiagen (17 genes)
P552	Negative	NGS: Qiagen (17 genes)
P553	Negative	NGS: Qiagen (17 genes)
P554	Negative	NGS: Qiagen (17 genes)
P555	Negative	NGS: Qiagen (17 genes)
P556	Positive	NGS: Qiagen (17 genes); MLPA
P557	Positive	NGS: Qiagen (17 genes)
P558	Positive	NGS: Qiagen (17 genes)

P559	Positive	NGS: Qiagen (17 genes)
P564	Negative	NGS: Qiagen (17 genes)
P565	Negative	NGS: Qiagen (17 genes)
P566	Negative	NGS: Qiagen (17 genes)
P567	Positive	NGS: Qiagen (17 genes)
P570	Positive	NGS: Qiagen (17 genes)
P574	Positive	NGS: Qiagen (17 genes)
P579	Negative	NGS: Qiagen (17 genes)
P581	Negative	NGS: Qiagen (17 genes)
P582	Negative	NGS: Qiagen (17 genes)
P583	Negative	NGS: Qiagen (17 genes)
P584	Negative	NGS: Qiagen (17 genes)
P585	Positive	NGS: Qiagen (17 genes); MLPA
P586	Positive	NGS: Qiagen (17 genes)
P587	Negative	NGS: Qiagen (17 genes)
P588	Negative	NGS: Qiagen (17 genes)
P589	Negative	NGS: Qiagen (17 genes)
P590	Negative	NGS: Qiagen (17 genes)
P591	Positive	NGS: Qiagen (17 genes)
P592	Negative	NGS: Qiagen (17 genes)
P593	Negative	NGS: Qiagen (17 genes)
P594	Negative	NGS: Qiagen (17 genes)
P596	Negative	NGS: Qiagen (17 genes)
P597	Negative	NGS: Qiagen (17 genes)
P598	Positive	NGS: Qiagen (17 genes)
P599	Negative	NGS: Qiagen (17 genes)
P600	Negative	NGS: Qiagen (17 genes)
P601	Positive	NGS: Qiagen (17 genes)
P606	Negative	NGS: Qiagen (17 genes)
P607	Negative	NGS: Qiagen (17 genes)
P608	Negative	NGS: Qiagen (17 genes)
P609	Negative	NGS: Qiagen (17 genes)
P610	Positive	NGS: Qiagen (17 genes)
P612	Negative	NGS: Qiagen (17 genes)
P614	Positive	NGS: Qiagen (17 genes)
P618	Negative	NGS: Qiagen (17 genes)
P619	Negative	NGS: Qiagen (17 genes)
P620	Negative	NGS: Qiagen (17 genes)
P621	Negative	NGS: Qiagen (17 genes)
P622	Negative	NGS: Qiagen (17 genes)
P623	Negative	NGS: Qiagen (17 genes)
P624	Negative	NGS: Qiagen (17 genes)
P625	Negative	NGS: Qiagen (17 genes)
P627	Negative	NGS: Qiagen (17 genes)
P628	Negative	NGS: Qiagen (17 genes)
P629	Positive	NGS: Qiagen (17 genes)
P630	Negative	NGS: Qiagen (17 genes)
P631	Negative	NGS: Qiagen (17 genes)
P632	Negative	NGS: Qiagen (17 genes)

P633	Negative	NGS: Qiagen (17 genes)
P634	Negative	NGS: Qiagen (17 genes)
P635	Negative	NGS: Qiagen (17 genes)
P636	Positive	NGS: Qiagen (17 genes)
P637	Negative	NGS: Qiagen (17 genes)
P638	Negative	NGS: Qiagen (17 genes)
P639	Positive	NGS: Qiagen (17 genes)
P640	Positive	NGS: Qiagen (17 genes)
P641	Positive	NGS: Qiagen (17 genes)
P643	Negative	NGS: TWIST (18 genes)
P644	Negative	NGS: TWIST (18 genes)
P647	Negative	NGS: TWIST (18 genes)
P648	Negative	NGS: TWIST (18 genes)
P649	Negative	NGS: TWIST (18 genes)
P650	Negative	NGS: TWIST (18 genes)
P651	Negative	NGS: TWIST (18 genes)
P652	Negative	NGS: TWIST (18 genes)
P656	Negative	NGS: TWIST (18 genes)
P657	Negative	NGS: TWIST (18 genes)
P658	Negative	NGS: TWIST (18 genes)
P659	Negative	NGS: TWIST (18 genes)
P660	Negative	NGS: TWIST (18 genes)
P661	Negative	NGS: TWIST (18 genes)
P662	Negative	NGS: TWIST (18 genes)
P663	Positive	NGS: TWIST (18 genes)
P664	Negative	NGS: TWIST (18 genes)
P665	Positive	NGS: TWIST (18 genes)
P666	Positive	NGS: TWIST (18 genes)
P669	Negative	NGS: TWIST (18 genes)
P670	Negative	NGS: TWIST (18 genes)
P671	Negative	NGS: TWIST (18 genes)
P672	Negative	NGS: TWIST (18 genes)
P673	Negative	NGS: TWIST (18 genes)
P674	Negative	NGS: TWIST (18 genes)
P675	Positive	NGS: TWIST (18 genes)
P676	Negative	NGS: TWIST (18 genes)
P677	Negative	NGS: TWIST (18 genes)
P678	Negative	NGS: TWIST (18 genes)
P679	Negative	NGS: TWIST (20 genes)
P680	Negative	NGS: TWIST (18 genes)
P681	Negative	NGS: TWIST (18 genes)
P682	Negative	NGS: TWIST (18 genes)
P683	Negative	NGS: TWIST (18 genes)
P684	Positive	NGS: TWIST (18 genes)
P685	Negative	NGS: TWIST (18 genes)
P686	Positive	NGS: TWIST (18 genes)
P689	Negative	NGS: TWIST (18 genes)
P690	Negative	NGS: TWIST (18 genes)
P691	Negative	NGS: TWIST (18 genes)

P692	Negative	NGS: TWIST (18 genes)
P693	Negative	NGS: TWIST (18 genes)
P694	Negative	NGS: TWIST (18 genes)
P695	Negative	NGS: TWIST (18 genes)
P696	Positive	NGS: TWIST (18 genes)
P697	Negative	NGS: TWIST (18 genes)
P698	Negative	NGS: TWIST (18 genes)
P699	Negative	NGS: TWIST (18 genes)
P700	Negative	NGS: TWIST (18 genes)
P701	Negative	NGS: TWIST (18 genes)
P702	Negative	NGS: TWIST (18 genes)
P703	Negative	NGS: TWIST (18 genes)
P704	Negative	NGS: TWIST (18 genes)
P706	Negative	NGS: TWIST (18 genes)
P707	Negative	NGS: TWIST (18 genes)
P708	Negative	NGS: TWIST (18 genes)
P709	Negative	NGS: TWIST (18 genes)
P710	Negative	NGS: TWIST (18 genes)
P711	Negative	NGS: TWIST (18 genes)
P712	Negative	NGS: TWIST (18 genes)
P713	Positive	NGS: TWIST (18 genes)
P715	Negative	NGS: TWIST (18 genes)
P717	Negative	NGS: TWIST (18 genes)
P718	Negative	NGS: Qiagen (17 genes)
P719	Negative	NGS: TWIST (18 genes)
P720	Negative	NGS: TWIST (18 genes)
P721	Negative	NGS: TWIST (18 genes)
P722	Positive	NGS: TWIST (18 genes)
P724	Negative	NGS: TWIST (18 genes)
P725	Negative	NGS: TWIST (18 genes)

Table S3. Clinical data of patients with *HNF1A* mutation.

Family ID	Sample ID	Age at Diagnosis of Diabetes	Age at Receiving Genetic dg	BMI*	Obesity	Complications	Therapy BEFORE Genetic Diagnosis	FPG (0') (mmol/L)	PPG (120') (mmol/L)	HbA1c % (mmol/mol)	MODY Calculator (%)	Family Screening
**F007**	**P020**	**11**	**29**	**22.3**	**no**	**N/A**	**insulin**	**N/A**	**N/A**	**7.8 (61.7)**	**75.5**	**multiple generations affected**
F007	P021	no diabetes	28	N/A	N/A	N/A	N/A	N/A	N/A	N/A	N/A	multiple generations affected
F007	P022	no diabetes	4	N/A	N/A	N/A	N/A	N/A	N/A	N/A	N/A	multiple generations affected
F007	P023	no diabetes	1	17	no	none	none	N/A	N/A	N/A	N/A	multiple generations affected
**F012**	**P032**	**N/A**	**35**	**25**	**no**	**retinopathy, IHD**	**insulin**	**8.6**	**12**	**13.4 (123.0)**	**N/A**	**multiple generations affected**
F012	P033	no diabetes	7	N/A	N/A	N/A	N/A	N/A	N/A	N/A	N/A	multiple generations affected
**F020**	**P049**	**25**	**47**	**25.4**	**no**	**none**	**insulin**	**7.9**	**9.9**	**8.2 (66.1)**	**45.5**	**multiple generations affected**
F020	P050	19	24	27.8	no	none	OAD - metformin	6.8	9.1	6.2 (43.3)	75.5	multiple generations affected
F020	P051	39	69	32.4	yes	retinopathy	OAD - metformin + insulin	7.7	9.8	7.5 (58.5)	N/A	multiple generations affected
F020	P053	no diabetes	24	24.5	no	none	N/A	N/A	N/A	N/A	N/A	multiple generations affected
**F025**	**P058**	**11**	**37**	**23**	**no**	**proteinuria, retinopathy**	**insulin**	**N/A**	**N/A**	**N/A**	**N/A**	**multiple generations affected**
F025	P059	13	16	27.6	yes	none	insulin	6.0	N/A	7.0 (53.0)	8.2	multiple generations affected
**F095**	**P174**	**15**	**44**	**26**	**no**	**none**	**insulin**	**N/A**	**N/A**	**7.1 (54.1)**	**6.4**	**multiple generations affected**
F095	P175	no diabetes	27	N/A	N/A	N/A	N/A	N/A	N/A	N/A	N/A	multiple generations affected
F095	P176	no diabetes	24	N/A	N/A	N/A	N/A	N/A	N/A	N/A	N/A	multiple generations affected
**F096**	**P177**	**12**	**12**	**16.9**	**no**	**none**	**insulin**	**14.8**	**N/A**	**N/A**	**N/A**	**no family members tested**
**F098**	**P186**	**12**	**12**	**19.4**	**no**	**none**	**insulin**	**6.7**	**13.6**	**6.3 (45.4)**	**49.4**	**multiple generations affected**
F098	P189	30	54	22.5	no	none	insulin	11.3	N/A	7.4 (57.4)	4.6	multiple generations affected

Family ID	Sample ID	Age at Diagnosis of Diabetes	Age at Receiving Genetic dg	BMI*	Obesity	Complications	Therapy BEFORE Genetic Diagnosis	FPG (0') (mmol/L)	PPG (120') (mmol/L)	HbA1c % (mmol/mol)	MODY Calculator (%)	Family Screening
**F104**	**P198**	**32**	**32**	**20.6**	**no**	**none**	**diet**	**7.81**	**11.86**	**6.2 (44.3)**	**75.5**	**no family members tested**
**F128**	**P229**	**13**	**17**	**24.1**	**no**	**none**	**insulin**	**8.8**	**N/A**	**8.7 (71.6)**	**1.9**	**no family members tested**
**F141**	**P250**	**33**	**37**	**N/A**	**N/A**	**N/A**	**diet**	**N/A**	**N/A**	**8.5 (69.4)**	**N/A**	**multiple generations affected**
F141	P252	16	39	N/A	N/A	N/A	OAD – sulphonylurea	N/A	N/A	N/A	N/A	multiple generations affected
F141	P253	42	79	N/A	N/A	N/A	OAD	N/A	N/A	N/A	N/A	multiple generations affected
**F155**	**P273**	**14**	**17**	**27.5**	**no**	**none**	**insulin**	**N/A**	**N/A**	**7.0 (53.0)**	**1.9**	**no family members tested**
**F159**	**P277**	**36**	**44**	**24.5**	**no**	**none**	**OAD – sulphonylurea**	**8.2**	**13.5**	**8.1 (65.0)**	**N/A**	**no family members tested**
**F204**	**P346**	**N/A**	**42**	**N/A**	**N/A**	**N/A**	**N/A**	**N/A**	**N/A**	**N/A**	**N/A**	**parents not tested**
**F228**	**P378**	**13**	**15**	**N/A**	**N/A**	**none**	**insulin**	**10.9**	**N/A**	**7.8 (61.7)**	**N/A**	**no family members tested**
**F234**	**P384**	**35**	**50**	**36.1**	**yes**	**none**	**OAD - metformin + sulphonylurea**	**7.0**	**N/A**	**7.1 (54.1)**	**4.6**	**no family members tested**
**F259**	**P418**	**13**	**13**	**23.6**	**no**	**N/A**	**N/A**	**6.1**	**15.9**	**6.5 (47.5)**	**N/A**	**multiple generations affected**
F259	P422	4	41	N/A	N/A	none	insulin	N/A	N/A	12.0 (107.7)	N/A	multiple generations affected
**F283**	**P447**	**N/A**	**13**	**N/A**	**N/A**	**N/A**	**N/A**	**N/A**	**N/A**	**N/A**	**N/A**	**no family members tested**
**F287**	**P451**	**N/A**	**31**	**N/A**	**N/A**	**N/A**	**N/A**	**N/A**	**N/A**	**N/A**	**N/A**	**no family members tested**
**F297**	**P461**	**12**	**17**	**31**	**yes**	**none**	**insulin**	**15.2**	**N/A**	**10.6 (92.4)**	**6.4**	**no family members tested**
**F302**	**P466**	**15**	**28**	**20.7**	**no**	**none**	**insulin**	**7.0**	**14.9**	**6.0 (42.1)**	**49.4**	**no family members tested**
**F333**	**P512**	**17**	**19**	**25.3**	**no**	**none**	**diet**	**4.6**	**8.9**	**5.2 (33.3)**	**75.5**	**multiple generations affected**
F333	P514	45	47	30.8	yes	acanthosis nigricans	OAD - metformin	10.2	N/A	6.9 (51.9)	N/A	multiple generations affected
**F361**	**P545**	**23**	**37**	**22.2**	**no**	**none**	**insulin**	**4.4**	**14.8**	**5.8 (39.9)**	**75.5**	**parents not tested**
**F366**	**P550**	**15**	**26**	**20.3**	**no**	**none**	**OAD – sulphonylurea**	**4.2**	**N/A**	**5.7 (38.8)**	**75.5**	**no family members tested**

Family ID	Sample ID	Age at Diagnosis of Diabetes	Age at Receiving Genetic dg	BMI*	Obesity	Complications	Therapy BEFORE Genetic Diagnosis	FPG (0') (mmol/L)	PPG (120') (mmol/L)	HbA1c % (mmol/mol)	MODY Calculator (%)	Family Screening
**F380**	**P567**	**17**	**20**	**24**	**no**	**N/A**	**insulin**	**N/A**	**N/A**	**5.4 (35.5)**	**49.4**	**multiple generations affected**
F380	P569	27	56	24	no	N/A	insulin	N/A	N/A	7.2 (55.2)	35.8	multiple generations affected
**F394**	**P591**	**13**	**32**	**19.7**	**no**	**retinopathy**	**OAD – sulphonylurea**	**6.71**	**N/A**	**N/A**	**N/A**	**no family members tested**
**F423**	**P629**	**N/A**	**16**	**N/A**	**N/A**	**N/A**	**OAD - metformin**	**N/A**	**N/A**	**N/A**	**N/A**	**parents not tested**
F423	P726	no diabetes	14	N/A	N/A	N/A	N/A	N/A	N/A	N/A	N/A	parents not tested
**F430**	**P636**	**10**	**10**	**12.9**	**no**	**N/A**	**diet**	**6.5**	**15.5**	**5.3 (34.4)**	**75.5**	**no family members tested**
**F452**	**P663**	**16**	**18**	**25.1**	**no**	**none**	**insulin**	**20**	**N/A**	**9.6 (81.4)**	**75.5**	**no family members tested**
**F474**	**P686**	**N/A**	**15**	**obese**	**yes**	**N/A**	**N/A**	**7.5**	**N/A**	**N/A**	**N/A**	**multiple generations affected**
F474	P688	no diabetes	37	N/A	N/A	N/A	N/A	N/A	N/A	N/A	N/A	multiple generations affected
**F508**	**P722**	**N/A**	**18**	**N/A**	**N/A**	**N/A**	**insulin**	**N/A**	**N/A**	**8.0 (63.9)**	**N/A**	**no family members tested**

Index patients are shown in bold. Age at diabetes: N/A—the patient has diabetes, but no information was received regarding the age of diagnosis; no diabetes - no information was given in the application form that the patient shows any signs of diabetes. dg—diagnosis; OAD—oral antidiabetic drug; IHD—Ischemic Heart Disease.
* BMI data refers to the time of referral for genetic testing.

Table S4. Clinical data of patients having a mutation in other MODY-causing genes.

Family ID	Sample ID	Age at Diagnosis of Diabetes	Age at Receiving Genetic dg	BMI*	Obesity	Complications	Therapy BEFORE dg	FPG (0') (mmol/L)	PPG (120') (mmol/L)	HbA1c % (mmol/mol)	MODY Calculator %	Family Screening
**F196**	**P331**	**32**	**39**	**20.3**	**no**	**none**	**OAD - metformin**	**8.8**	**N/A**	**8.2 (66.1)**	**35.8**	**multiple generations affected**
F196	P332	35	67	28.7	no	retinopathy, nephropathy, uraemia, PAD, proteinuria, IHD, CHF	insulin	6.9	N/A	5.8 (39.9)	4.6	multiple generations affected
F196	P334	17	42	26.8	no	retinopathy	insulin	9.2	N/A	6.7 (49.7)	8.2	multiple generations affected
**F305**	**P469**	**15**	**45**	**30.7**	**yes**	**retinopathia, proteinuria**	**insulin**	**N/A**	**N/A**	**11.0 (96.7)**	**58**	**no family members tested**
**F076**	**P150**	**N/A**	**N/A**	**N/A**	**N/A**	**N/A**	**N/A**	**N/A**	**N/A**	**N/A**	**N/A**	**no family members tested**

Family ID	Sample ID	Age at Diagnosis of Diabetes	Age at Receiving Genetic dg	BMI*	Obesity	Complications	Therapy BEFORE dg	FPG (0') (mmol/L)	PPG (120') (mmol/L)	HbA1c % (mmol/mol)	MODY Calculator %	Family Screening
**F091**	**P168**	**15**	**16**	**27.9**	**no**	**renal cysts**	**OAD - metformin**	**N/A**	**N/A**	**8.7 (71.6)**	**75.5**	**siblings positive, parents not tested**
F091	P169	no diabetes	19	N/A	N/A	N/A	N/A	N/A	N/A	N/A	N/A	siblings positive, parents not tested
**F172**	**P297**	**15**	**17**	**29.5**	**yes**	**N/A**	**diet**	**6.1**	**13.9**	**6.7 (49.7)**	**75.5**	**no family members tested**
**F372**	**P556**	**31**	**33**	**23.9**	**no**	**proteinuria, renal cysts**	**insulin**	**8.2**	**N/A**	**8.6 (70.5)**	**21**	**no family members tested**
**F388**	**P585**	**27**	**38**	**N/A**	**N/A**	**none**	**OAD - sulphonylurea**	**6.9**	**11**	**7.7 (60.7)**	**N/A**	**no family members tested**
**F097**	**P178**	**26**	**42**	**22**	**no**	**none**	**insulin**	**N/A**	**N/A**	**about 6**	**49.4**	**multiple generations affected**
F097	P179	10	17	19	no	none	insulin	N/A	N/A	8 - 9	75.5	multiple generations affected
**F192**	**P324**	**10**	**16**	**28**	**yes**	**none**	**diet**	**8.2**	**13.3**	**6.6 (48.6)**	**75.5**	**no family members tested**
**F284**	**P448**	**5**	**8**	**N/A**	**N/A**	**N/A**	**N/A**	**N/A**	**N/A**	**7.6 (59.6)**	**N/A**	**no family members tested**
**F315**	**P483**	**13**	**16**	**18.4**	**no**	**polyuria, polydipsia**	**none**	**6.7**	**9.4**	**6.6 (48.6)**	**75.5**	**multiple generations affected**
F315	P485	43	44	23.7	no	none	diet	6 -7	7.9	7.1 (54.1)	N/A	multiple generations affected

Index patients are shown in bold. Age at diabetes: N/A—the patient has diabetes, but no information was received regarding the age of diagnosis; no diabetes—no information was given in the application form that the patient shows any signs of diabetes. dg—diagnosis; OAD—oral antidiabetic drug; PAD—Peripheral Arterial Disease; IHD—Ischemic Heart Disease; CHF—Congestive Heart Failure. * BMI data refers to the time of referral for genetic testing.
